# CSI-GaitCov: A Wi-Fi channel state information dataset for gait-based person identification across walking-direction and backpack-carrying covariates

**DOI:** 10.1016/j.dib.2026.113303

**Published:** 2026-10-01

**Authors:** Nakib Aman, Durjoy Kumar Dutta, Md. Abdul Wadud, Al Arian Ahmad

**Affiliations:** Department of Computer Science and Engineering, Pabna University of Science and Technology, Pabna, 6600, Bangladesh

**Keywords:** Device-free sensing, Human biometrics, Wireless sensing, Intel AX210, Signal processing, Representation learning, Cross-condition evaluation

## Abstract

This data article presents CSI-GaitCov, a structured Wi-Fi Channel State Information (CSI) dataset developed to support gait-based person identification under controlled walking-direction and backpack-carrying covariates. The dataset was collected from 25 anonymized male participants aged 25–40 years in an indoor laboratory environment using a TP-Link Archer C6 V2.80 (AC1200) access point and an Intel AX210 Wi-Fi 6 network interface card with the PicoScenes CSI acquisition platform. Each participant completed 100 walking trials of approximately 6 s, yielding a total of 2,500 recordings. The protocol included 80 normal-walking and 20 backpack-carrying trials per participant, with walking direction balanced between left-to-right and right-to-left trajectories; the backpack condition involved an approximate load of 1 kg. CSI was acquired using a 2 × 2 MIMO configuration with an 80 MHz channel bandwidth and 245 retained CSI tones, providing four CSI channel sequences for each valid packet. The original packet-level recordings are released as CSV files containing timestamps, received signal strength indicator (RSSI), acquisition-configuration metadata, and serialized interleaved in-phase and quadrature CSI values, while a corresponding processed NPZ representation is provided for every trial. During preprocessing, valid complex CSI coefficients were reconstructed, transformed to log-amplitude, and temporally standardized onto a uniform 512-sample grid, producing fixed-size CSI arrays of 4 × 512 × 245 with aligned time and RSSI sequences. The release further includes a complete trial manifest, data dictionary, predefined benchmark splits, a 46-dimensional four-stream descriptive feature table, technical-validation summaries, benchmark results, and a reproducible processing and evaluation notebook. All 2500 raw trials were successfully processed, with a mean rejected-packet proportion of 0.0675%. CSI-GaitCov can support research on Wi-Fi sensing, gait biometrics, person identification, CSI preprocessing and representation learning, feature extraction, covariate-aware evaluation, and the development of robust wireless human-sensing methods.

Specifications Table

**Table:** 

Subject	Computer Science; Applied Machine Learning; Signal Processing; Computer Networks and Communications
Specific subject area	Wi-Fi CSI-based human sensing for gait biometrics and person identification under walking-direction and backpack-carrying covariates
Type of Data	Raw CSI data (.csv); Processed CSI data (.npz); Metadata and validation data (.csv)
Data collection	Wi-Fi CSI was collected in an indoor laboratory environment from 25 participants during 2,500 walking trials. A TP-Link Archer C6 V2.80 (AC1200) router served as the transmitter, and a laptop equipped with an Intel AX210 Wi-Fi 6 NIC served as the receiver. CSI was acquired using PicoScenes [1] under a 2 × 2 MIMO configuration with 80 MHz bandwidth and 245 retained subcarriers. Trials were performed in left-to-right and right-to-left directions under normal walking and approximately 1 kg backpack-carrying conditions.
Data source location	Institution: Pabna University of Science and Technology, Department of Computer Science and Engineering City/Region: Pabna-6600 Country: Bangladesh GPS coordinates: approximately 24.0131° N, 89.2794° E
Data accessibility	Repository name: Zenodo Dataset DOI: 10.5281/zenodo.22757555 Direct URL: https://doi.org/10.5281/zenodo.22757555
Related research article	None

## 1 Value of the Data

• The dataset provides Wi-Fi channel state information from 25 participants across 2,500 gait trials, with controlled variation in walking direction and backpack-carrying condition. This structure enables investigation of person-identification methods under both standard and covariate-varying walking scenarios.
• Raw CSI recordings are provided in .csv format together with processed CSI representations in .npz format, allowing researchers to work either from the original measurements or from a standardized trial-level representation suitable for signal analysis and machine-learning workflows.
• Each trial contains CSI acquired under a 2 × 2 MIMO configuration with 245 retained subcarriers, providing four CSI channel sequences per packet. The data can support studies of temporal, subcarrier-level, and multi-link characteristics of Wi-Fi signals associated with human gait.
• The inclusion of normal walking and approximately 1 kg backpack-carrying trials, together with left-to-right and right-to-left walking directions, allows reuse of the dataset for evaluating sensitivity to carrying-condition and directional covariates in gait-based wireless sensing.
• The accompanying metadata, benchmark split definitions, derived descriptive features, and technical-validation files facilitate reproducible evaluation, comparison of preprocessing or feature-extraction methods, and development of classical machine-learning and deep-learning approaches for Wi-Fi CSI-based human sensing.

## 2 Background

Wi-Fi sensing repurposes conventional wireless communication signals for passive human sensing by exploiting variations in radio propagation caused by human presence and movement [2]. Among the available wireless measurements, Channel State Information (CSI) provides fine-grained subcarrier-level information about the wireless channel and has been increasingly used for device-free human activity recognition, localization, and other forms of contactless human sensing [3]. In gait-based applications, coordinated motion of the torso and limbs produces characteristic temporal variations in CSI, allowing walking patterns to be analyzed without cameras, wearable devices, or direct physical contact [4]. However, CSI-based gait signatures are not determined solely by person-specific motion characteristics, since changes in walking direction can alter the observed wireless response [4]. Robust passive person-identification studies have further highlighted the importance of maintaining identity-related information under variations in gait observations and sensing conditions [5]. More broadly, the sensitivity of CSI measurements to environmental and domain changes has motivated increasing attention toward cross-domain robustness and generalization in Wi-Fi sensing systems [6]. Recent CSI-based gait research has also investigated multi-view feature representations and few-sample learning strategies to improve identity recognition under changing or limited observations [7]. Recent work has further demonstrated device-free gait identification using ESP32-based Wi-Fi CSI with multi-view signal representations and deep learning, highlighting the feasibility of low-cost embedded CSI sensing for gait biometrics [8]. Within this context, CSI-GaitCov was compiled to provide repeated Wi-Fi CSI gait measurements from the same participants under systematically controlled covariates. The dataset includes left-to-right and right-to-left walking together with normal and backpack-carrying conditions in a controlled indoor laboratory environment, providing a reusable resource for gait-based person identification, CSI signal-processing and representation studies, and evaluation under direction- and carrying-related covariate changes.

## 3 Data Description

CSI-GaitCov contains 2,500 trial-level Wi-Fi Channel State Information (CSI) recordings collected from 25 anonymized participants under controlled indoor walking conditions. Each participant contributed 100 trials, comprising 80 normal-walking trials and 20 backpack-carrying trials. Walking direction was balanced within each participant, with 50 left-to-right (L→R) and 50 right-to-left (R→L) trials. Overall, the dataset contains 2,000 normal-walking trials and 500 backpack-carrying trials, with 1,250 trials recorded in each walking direction. The backpack condition involved a regular school backpack with an approximate load of 1 kg. Each trial represents approximately 6 s of walking activity, and participant identity is represented using anonymous identifiers S01 through S25. The participant-wise and aggregate trial distributions are shown in Fig. 1, while the complete trial composition is summarized in Table 1.

**Fig. 1:**
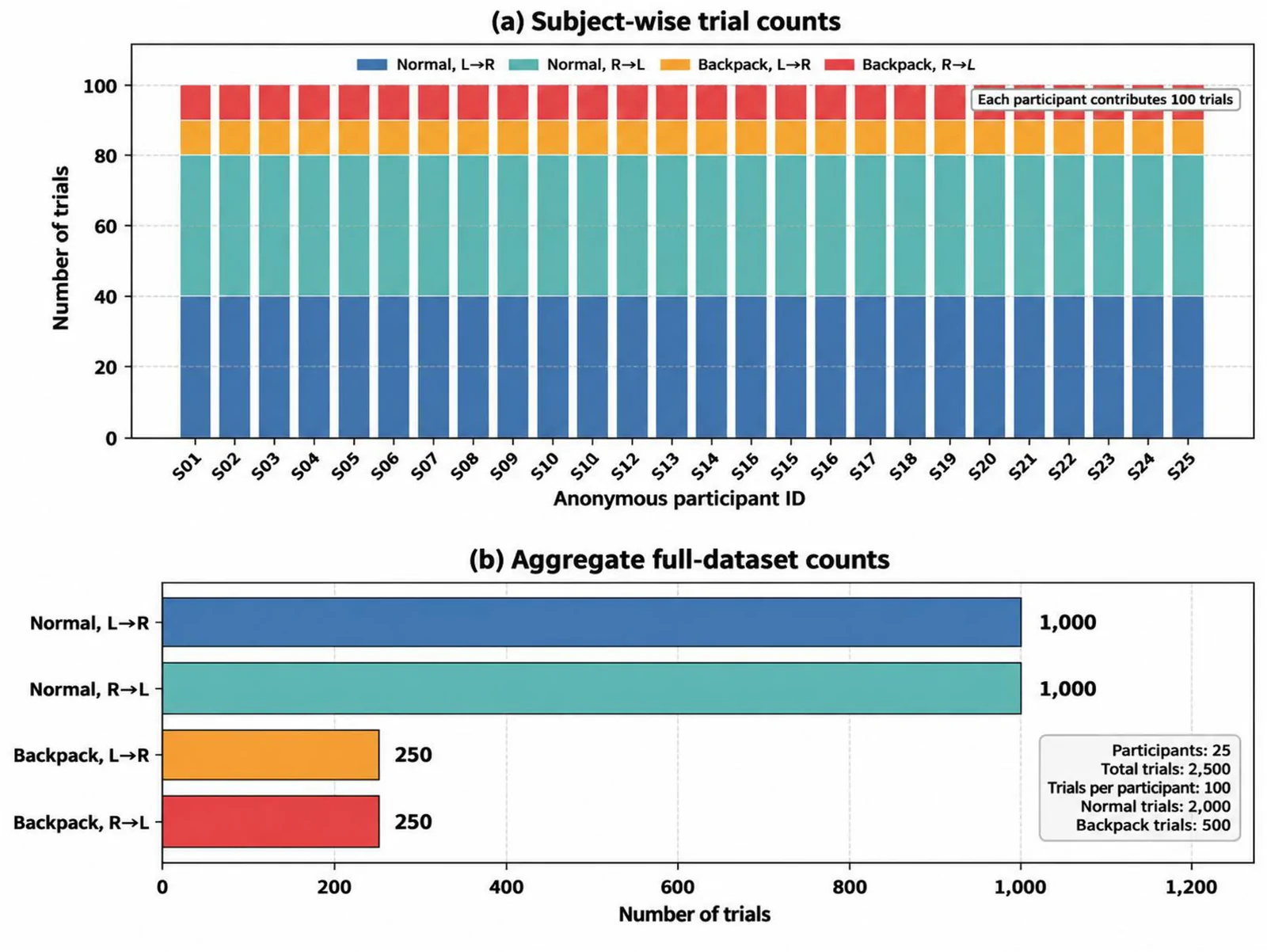
Participant-wise and aggregate trial distribution of CSI-GaitCov. (a) Trial counts for the 25 anonymized participants across the four direction–carrying combinations. (b) Aggregate counts for normal and backpack walking in both directions.

**Table 1:** Composition of the CSI-GaitCov trial collection.

Dataset component	Per participant	Complete dataset
All trials	100	2,500
Normal walking	80	2,000
Backpack carrying	20	500
L→R direction	50	1,250
R→L direction	50	1,250
Normal, L→R	40	1,000
Normal, R→L	40	1,000
Backpack, L→R	10	250
Backpack, R→L	10	250

Each trial is released as one raw comma-separated values (.csv) file and, in the processed release, as a corresponding NumPy compressed archive (.npz) with the same filename stem. Filenames encode the anonymous participant identifier, trial number, walking direction, and carrying condition. For example, S02_T01_L_R_normal.csv denotes participant S02, trial 01, left-to-right walking under the normal condition, whereas S02_T01_L_R_normal.npz denotes the corresponding processed file. The naming convention is summarized in Table 2.

**Table 2:** Naming convention for trial-level files in CSI-GaitCov.

Component	Represents	Values	Example
Sxx	Participant ID	S01–S25	S02
Tyy	Trial number	T01–T100	T01
Direction	Walking direction	L_R or R_L	L_R
Condition	Carrying condition	normal or bag	normal
File extension	Data format	.csv or .npz	.csv

The public release is organized into four top-level directories: 01_Data, 02_Metadata, 03_Code_and_Validation, and 04_Documentation. The 01_Data directory contains the primary signal recordings in separate RAW and PROCESSED branches, with participant-specific folders from S01 through S25. The 02_Metadata directory contains the dataset manifest, data dictionary, benchmark split definitions, and derived four-stream features. Reproducibility resources and validation outputs are provided in 03_Code_and_Validation, while 04_Documentation contains supporting documentation, licensing information, and file-integrity records. The overall repository organization is illustrated in Fig. 2.

**Fig. 2:**
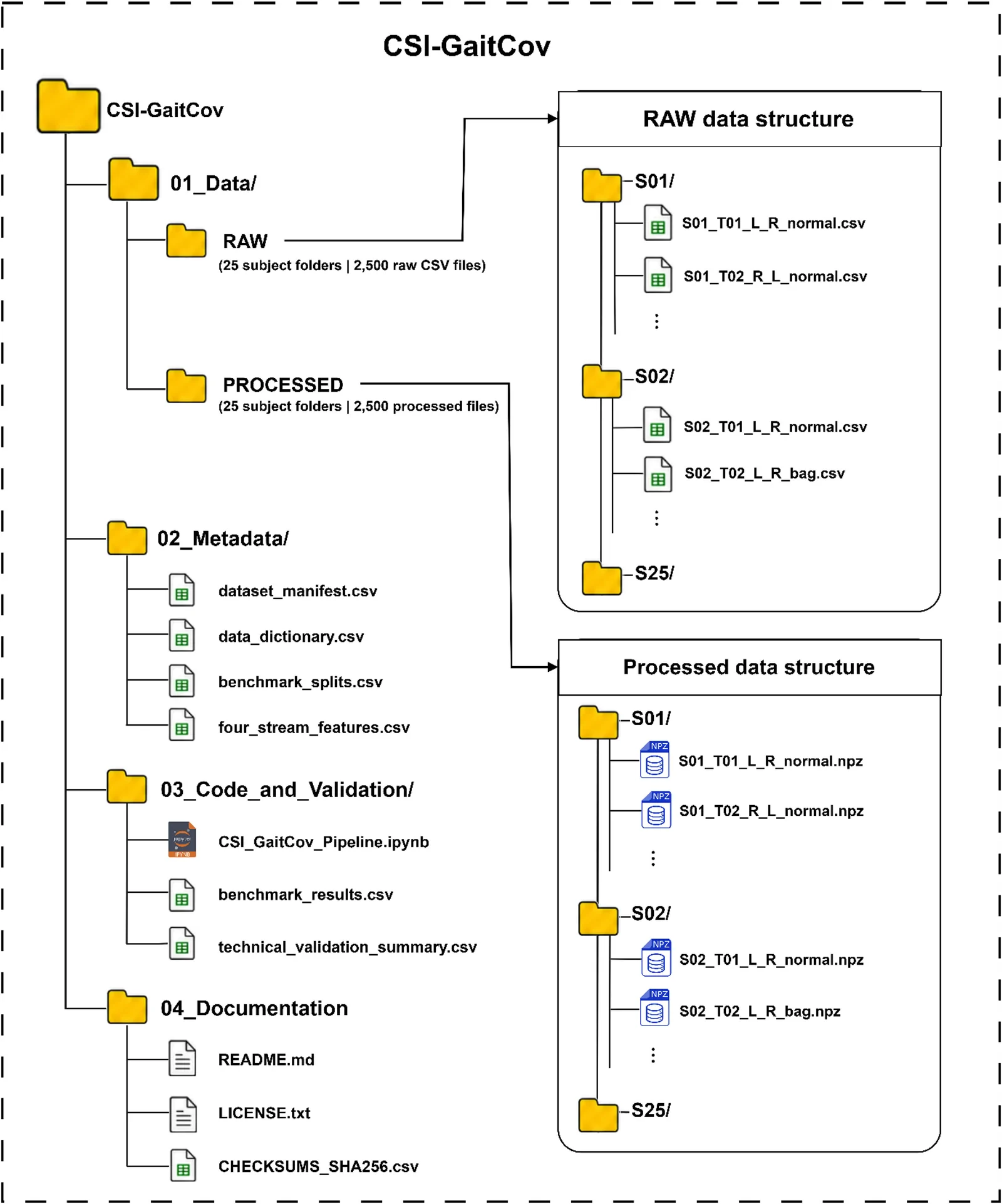
Repository organization of CSI-GaitCov, comprising four top-level directories for data, metadata, code and validation resources, and documentation.

The raw CSV files preserve packet-level measurements exported from PicoScenes. Each row corresponds to one captured Wi-Fi packet and contains packet timing, received signal strength, acquisition-configuration descriptors, and a serialized CSI payload. The fields contained in each raw trial file are listed in Table 3. For the retained acquisition configuration, valid packets contain 245 CSI tones under a 2 × 2 MIMO configuration. The serialized data field contains 1,960 numerical values, corresponding to 245 tones × 2 receive channels × 2 transmit channels × 2 components for the in-phase and quadrature values.

**Table 3:** Structure and description of fields in each raw trial-level CSV file.

Field	Description	Role in the released data
ts_sec	Packet timestamp in seconds	Primary temporal coordinate for packet ordering and trial-time reconstruction.
systemns	System timestamp in nanoseconds	Auxiliary acquisition-time information retained from the logger output.
rssi	Received signal strength indicator	Packet-level received-signal-strength measurement.
packetFormat	PicoScenes packet-format descriptor	Records the packet format reported during acquisition.
cbw	Channel bandwidth descriptor	Identifies the retained 80 MHz acquisition configuration.
numTones	Number of CSI tones/subcarriers	Expected value is 245 for retained packets.
numRx	Number of receive channels	Expected value is 2.
numTx	Number of transmit channels	Expected value is 2.
data_len	Length of the serialized CSI payload	Expected value is 1,960 for the retained configuration.
data	Serialized interleaved in-phase and quadrature values	Numerical payload from which complex CSI coefficients are reconstructed.

The interleaved in-phase and quadrature values contained in the data field represent the complex CSI coefficients associated with each valid packet. Under the 2 × 2 MIMO configuration, four CSI channel sequences are available per packet, each containing measurements over 245 subcarriers. These sequences are referred to as Stream 1, Stream 2, Stream 3, and Stream 4 throughout the released dataset. Generic stream terminology is used because the physical transmitter-receiver antenna-pair ordering of the flattened CSI payload was not independently verified; therefore, specific Tx-Rx antenna labels are not assigned to these sequences.

A processed NPZ counterpart is provided for every released trial to supply a standardized representation for direct computational reuse. Each processed file contains three arrays: x_logamp, t_sec, and rssi, as summarized in Table 4. The x_logamp array contains the log-amplitude CSI representation for the four channel sequences over 512 temporal samples and 245 subcarriers. The t_sec array contains the corresponding relative-time coordinate, while the rssi array contains received-signal-strength values aligned to the same temporal grid. The raw packet-level representation remains available independently of these processed arrays.

**Table 4:** Structure of the processed NPZ trial files in CSI-GaitCov.

Array	Shape	Data type	Description
x_logamp	4 × 512 × 245	float32	Log-amplitude CSI arranged as Stream × Time × Subcarrier.
t_sec	512	float32	Relative-time samples associated with the standardized trial grid.
rssi	512	float32	RSSI values aligned to the same 512-sample temporal grid.

Representative raw CSI recordings from the same anonymized participant are shown in Fig. 3 for the four combinations of walking direction and carrying condition: normal L→R, normal R→L, backpack L→R, and backpack R→L. In each panel, the four generic CSI channel sequences are concatenated along the CSI-coefficient axis, while the vertical axis represents log(1 + |CSI|). Time is derived from the original packet timestamps.

**Fig. 3:**
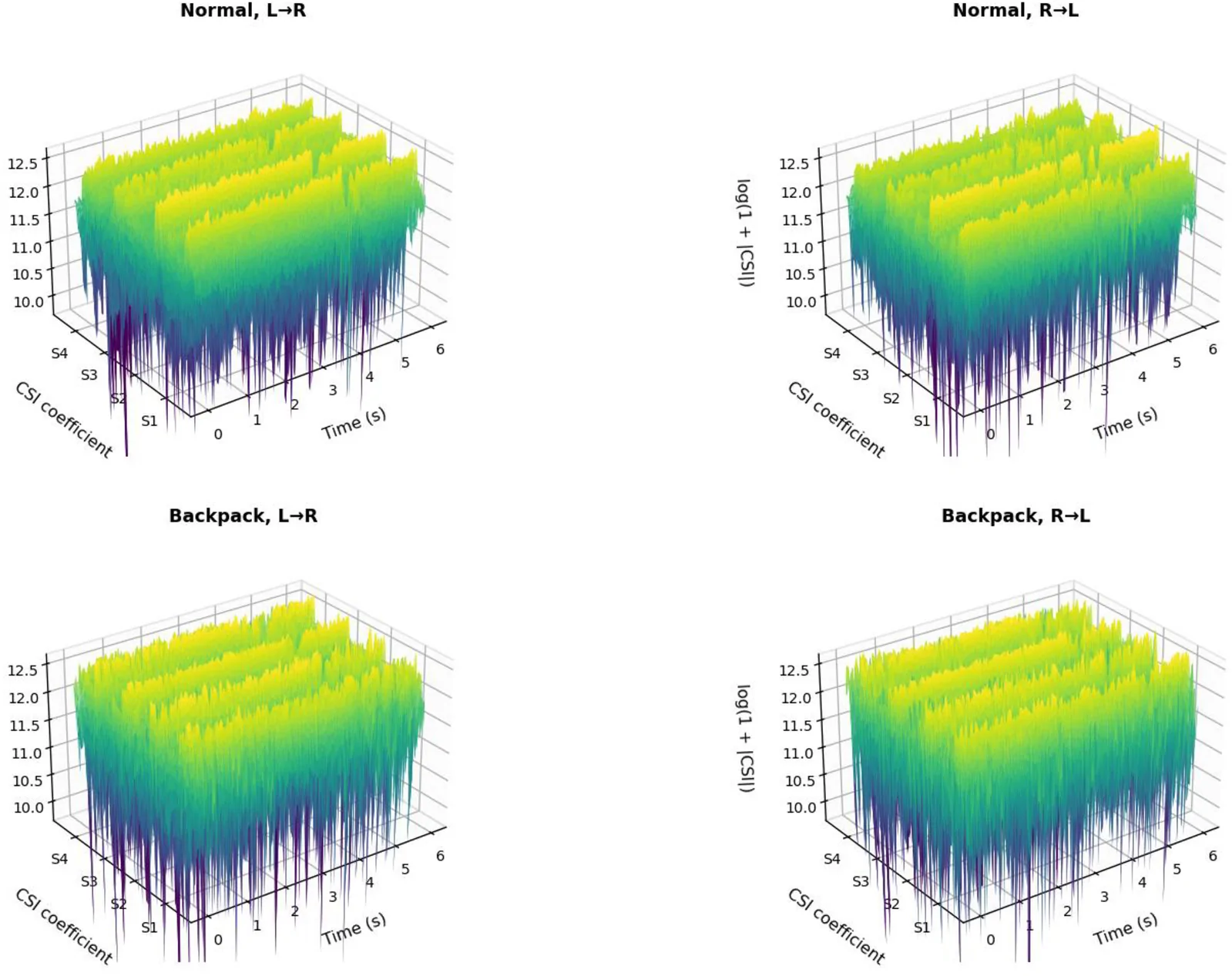
Representative raw CSI log-amplitude recordings across the four walking-direction and backpack conditions for the same anonymized participant.

The dataset_manifest.csv file provides the trial-level index for the complete collection and links each recording with its participant identifier, trial number, walking direction, carrying condition, and corresponding released files. The accompanying data_dictionary.csv documents the meaning of the released fields, labels, and arrays. The benchmark_splits.csv file contains the predefined trial assignments used to reproduce the released standard closed-set and strict covariate-shift evaluation protocols.

The four_stream_features.csv file contains 46 derived descriptive features for every trial. Ten descriptors are provided separately for each of the four CSI streams: mean log-amplitude, standard deviation, median, interquartile range, dynamic root-mean-square magnitude, motion root-mean-square magnitude, mean absolute temporal difference, temporal range, dominant frequency, and spectral entropy. Six additional columns contain pairwise correlations between the four streams. These values are distributed as derived descriptive data alongside, rather than in place of, the primary raw CSV and processed NPZ recordings.

The 03_Code_and_Validation directory contains CSI_GaitCov_Pipeline.ipynb, benchmark_results.csv, and technical_validation_summary.csv. The notebook documents the computational pipeline associated with the released data, while the two CSV files contain the tabulated benchmark outputs and consolidated technical-validation quantities. The 04_Documentation directory contains README.md, LICENSE.txt, and CHECKSUMS_SHA256.csv, providing repository usage information, licensing terms, and cryptographic hashes for file-integrity verification, respectively. The public dataset uses only the anonymous participant identifiers S01–S25 and does not contain participant names, facial information, video recordings, or other directly identifying visual information.

Detailed information on data acquisition and processing is provided in the Experimental Design, Materials and Methods section, while data-quality assessment, signal characterization, and benchmark results are reported separately in the Technical Validation section.

## 4 Experimental Design, Materials and Methods

CSI-GaitCov was developed through a controlled workflow comprising indoor Wi-Fi CSI acquisition, trial-level segmentation, packet validation, CSI reconstruction, temporal standardization, processed-data generation, derived feature extraction, and technical validation. The original packet-level CSV recordings were preserved without modification, while subsequent processing was performed separately to obtain standardized trial representations suitable for reproducible analysis. Packets inconsistent with the retained acquisition configuration were excluded during processing, after which the interleaved in-phase and quadrature samples were reconstructed as complex CSI. The resulting signals were converted to log-amplitude and mapped onto a uniform temporal grid before being stored as processed trial-level arrays. Dataset quality and analytical usability were subsequently assessed using packet- and trial-level quality measures together with reproducible baseline benchmarks. The complete data-generation and validation workflow is summarized in Fig. 4.

**Fig. 4:**
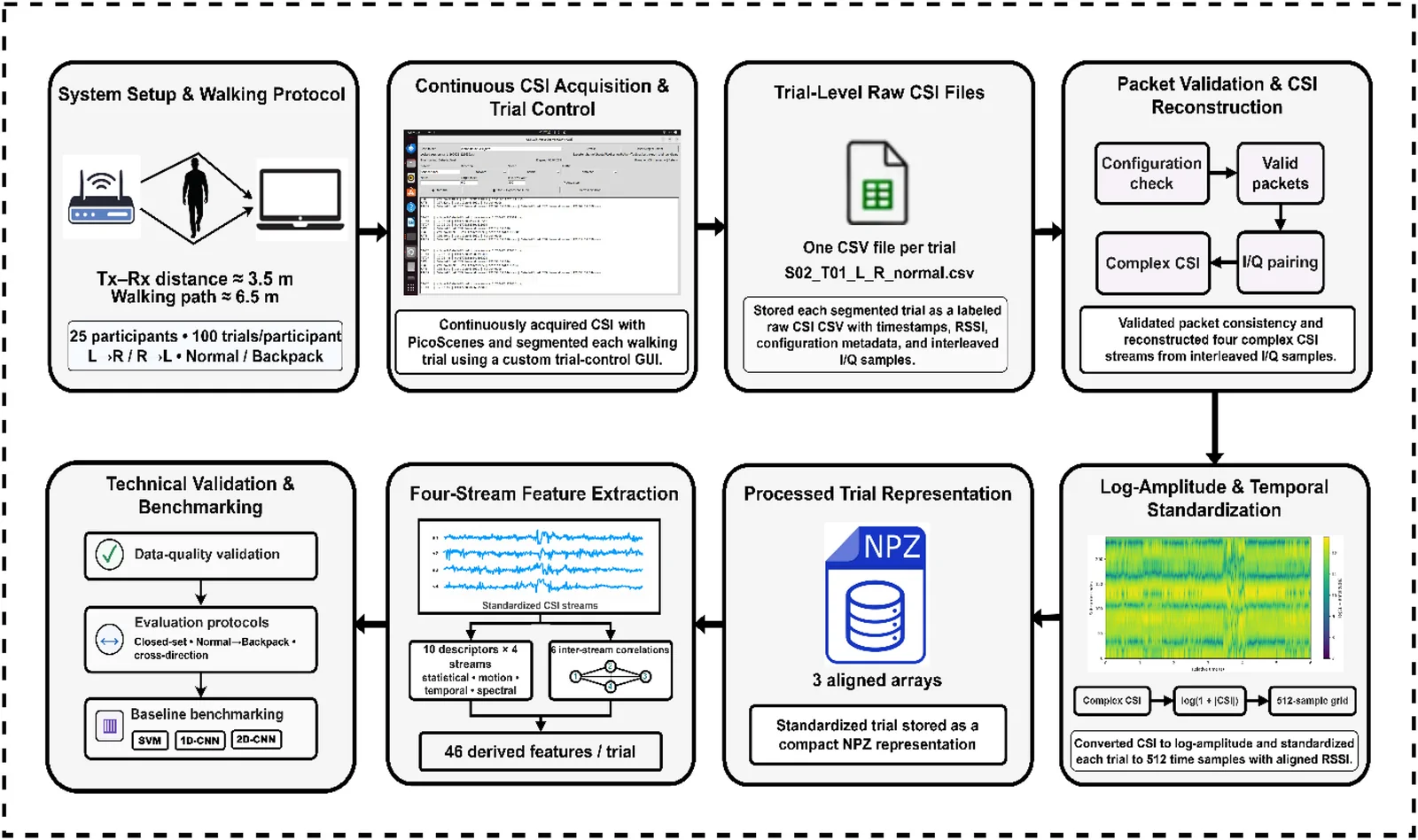
Overall CSI-GaitCov acquisition, processing, and validation workflow.

### 4.1 Experimental setup and sensing environment

Data collection was carried out in an indoor laboratory of the Department of Computer Science and Engineering, Pabna University of Science and Technology, Pabna, Bangladesh. The room measured approximately 8 m × 7 m × 3.5 m and retained its usual structural and furnishing elements during collection. A fixed transmitter-receiver geometry was maintained throughout the experiment: the transmitter (TX) and receiver (RX) were separated by approximately 3.5 m, and the designated walking route extended for approximately 6.5 m through the sensing region. Only one participant occupied the walking area during an individual trial. The TX/RX positions, walking trajectory, and major room layout were kept unchanged across participants and conditions. The environment, sensing geometry, and representative normal and backpack walking conditions are shown in Fig. 5.

**Fig. 5:**
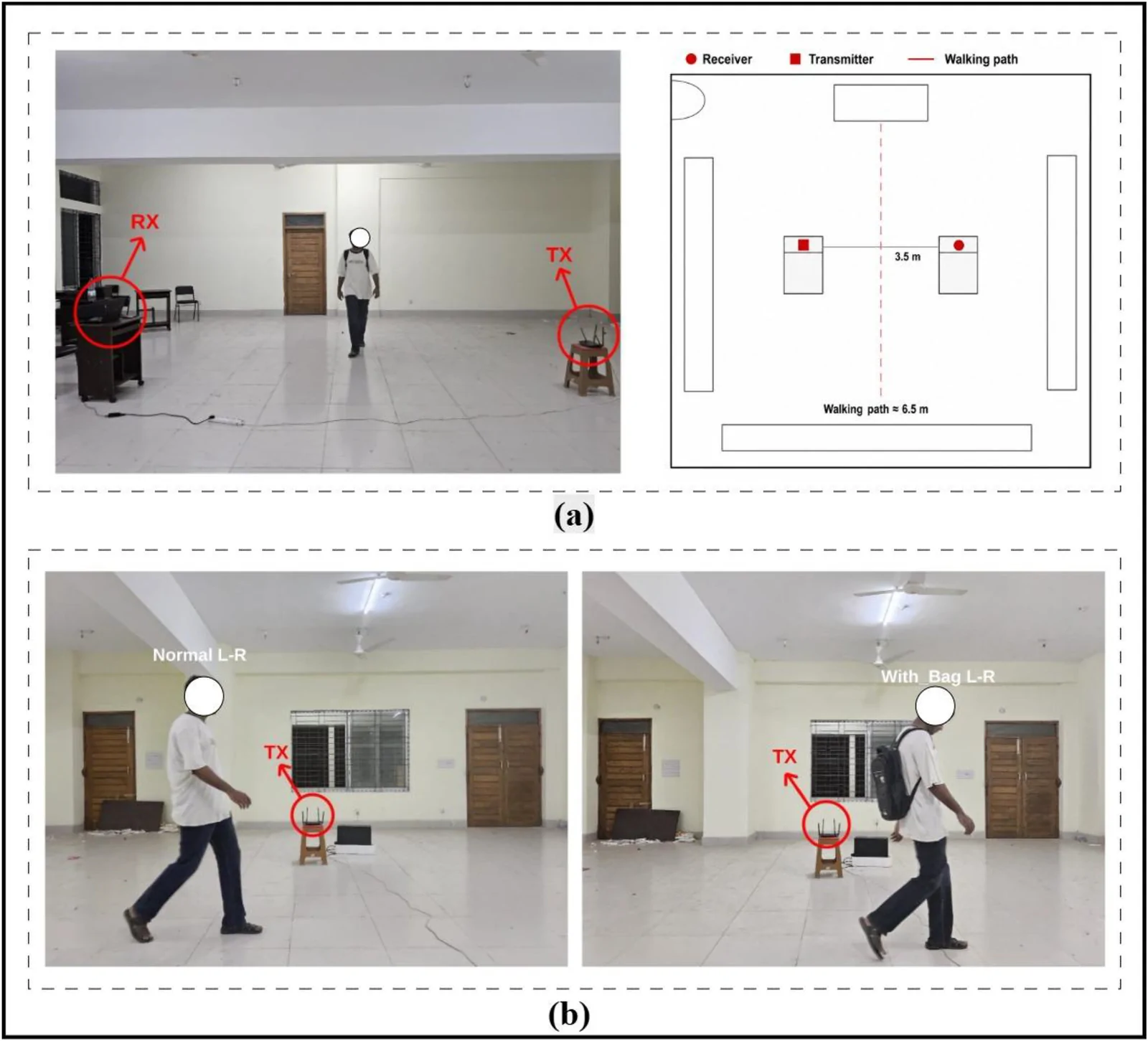
Experimental environment and walking protocol: (a) sensing layout; (b) normal and backpack walking conditions.

A TP-Link Archer C6 V2.80 (AC1200) router served as the wireless access point and transmitter. CSI was received on a Walton Passion BX5800 laptop equipped with an Intel AX210 Wi-Fi 6 network interface card. PicoScenes [1] provided continuous CSI acquisition under a 2 × 2 MIMO configuration. Packets retained for processing used an 80 MHz channel bandwidth and 245 CSI tones. Although the access point was configured for automatic channel selection, examination of the original PicoScenes acquisition metadata confirmed that the center frequency remained fixed at 5775 MHz across all 2,500 trials. No center-frequency hopping was observed during the data-collection campaign. Because the physical antenna-pair ordering of the flattened CSI payload was not independently verified, the four channel sequences are reported consistently as Stream 1–Stream 4. The hardware arrangement and conceptual 2 × 2 MIMO configuration are shown in Fig. 6.

**Fig. 6:**
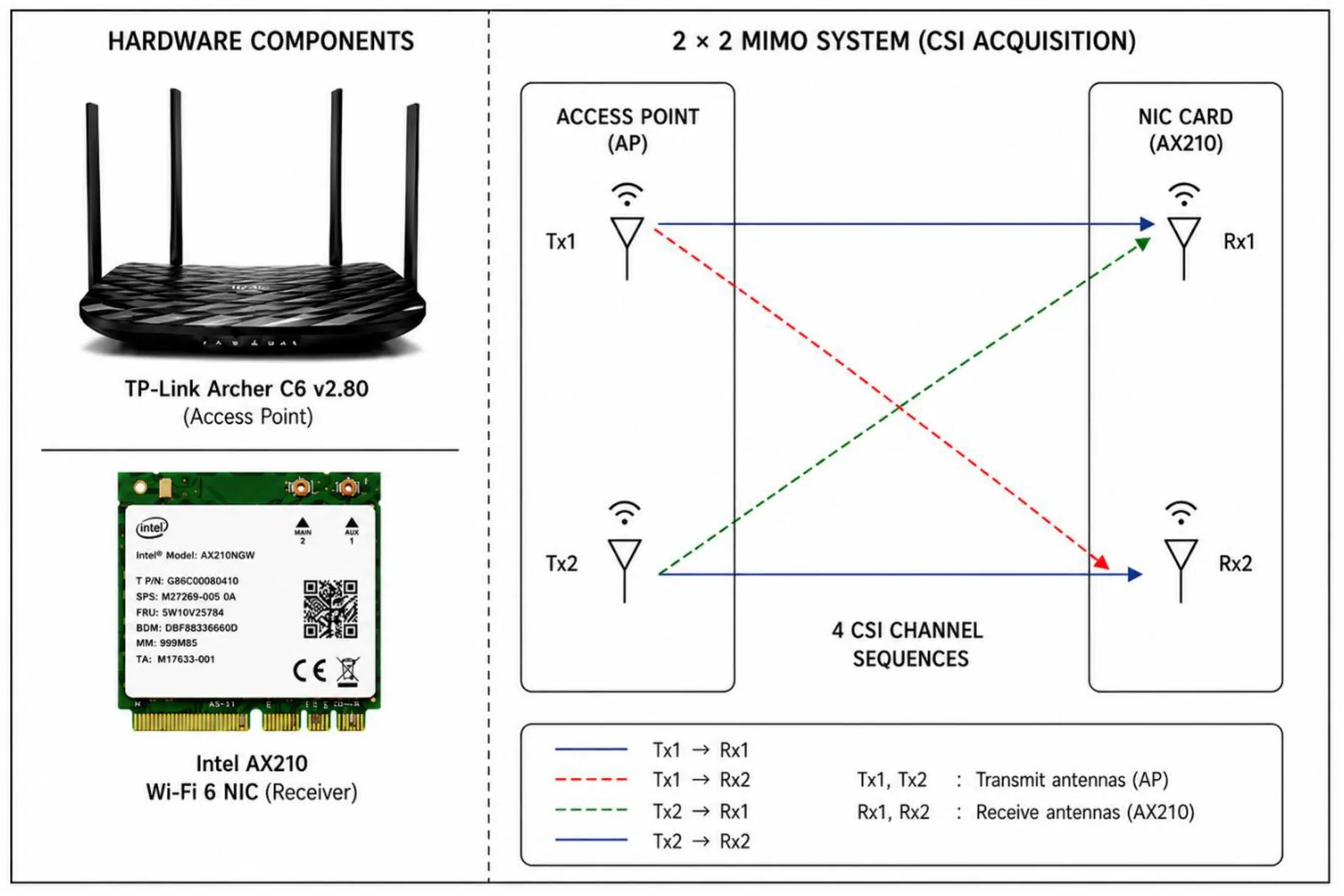
CSI acquisition hardware and conceptual 2 × 2 MIMO configuration; released CSI sequences are denoted as Stream 1–Stream 4.

### 4.2 Participants and walking protocol

A total of 25 male participants voluntarily took part in the CSI gait data-collection campaign. Participants were between 25 and 40 years of age, with heights ranging from 165 to 178 cm and body weights ranging from 56 to 95 kg. To preserve privacy, each participant was assigned an anonymous identifier from S01 to S25, and no personally identifying information was included in the released dataset. The recorded demographic attributes were used only to describe the participant cohort and were not incorporated as input variables in the preprocessing pipeline or baseline identification models. All participants were informed about the data-collection procedure before participation, and signed informed consent was obtained prior to recording. During acquisition, participants were instructed to walk naturally along the predefined path rather than deliberately modifying their stride length, walking rhythm, or body posture.

Each participant completed 100 walking trials, resulting in 2,500 trials across the full dataset. The protocol comprised 80 normal-walking trials and 20 backpack-carrying trials per participant. Walking direction was balanced within each participant: the normal condition contained 40 L→R and 40 R→L trials, while the backpack condition contained 10 L→R and 10 R→L trials. For the backpack condition, participants wore a regular backpack carrying an approximate load of 1 kg. Only one participant occupied the sensing region during each recording, and the same predefined walking path was followed at a natural, self-selected pace. Each acquisition targeted approximately 6 s of walking activity. The transmitter and receiver positions, walking trajectory, and principal environmental arrangement were kept unchanged throughout the collection campaign.

### 4.3 CSI acquisition, trial segmentation, and labeling

PicoScenes operated as a continuous CSI acquisition session rather than producing independent walking trials directly. A custom Python Tkinter-based graphical interface was therefore developed to delimit, label, and export individual walking trials from the continuous stream. Before each walk, the anonymous participant identifier, walking direction, carrying condition, and target trial duration were selected in the interface. The Start Trial control marked the beginning of the target interval, while Stop + Export closed the interval and exported the corresponding packet records as a single trial-level CSV file. Session status, elapsed time, and export information were displayed during acquisition, helping maintain consistent trial boundaries, labels, and filenames without repeated manual editing. The trial-control interface used during acquisition is shown in Fig. 7.

**Fig. 7:**
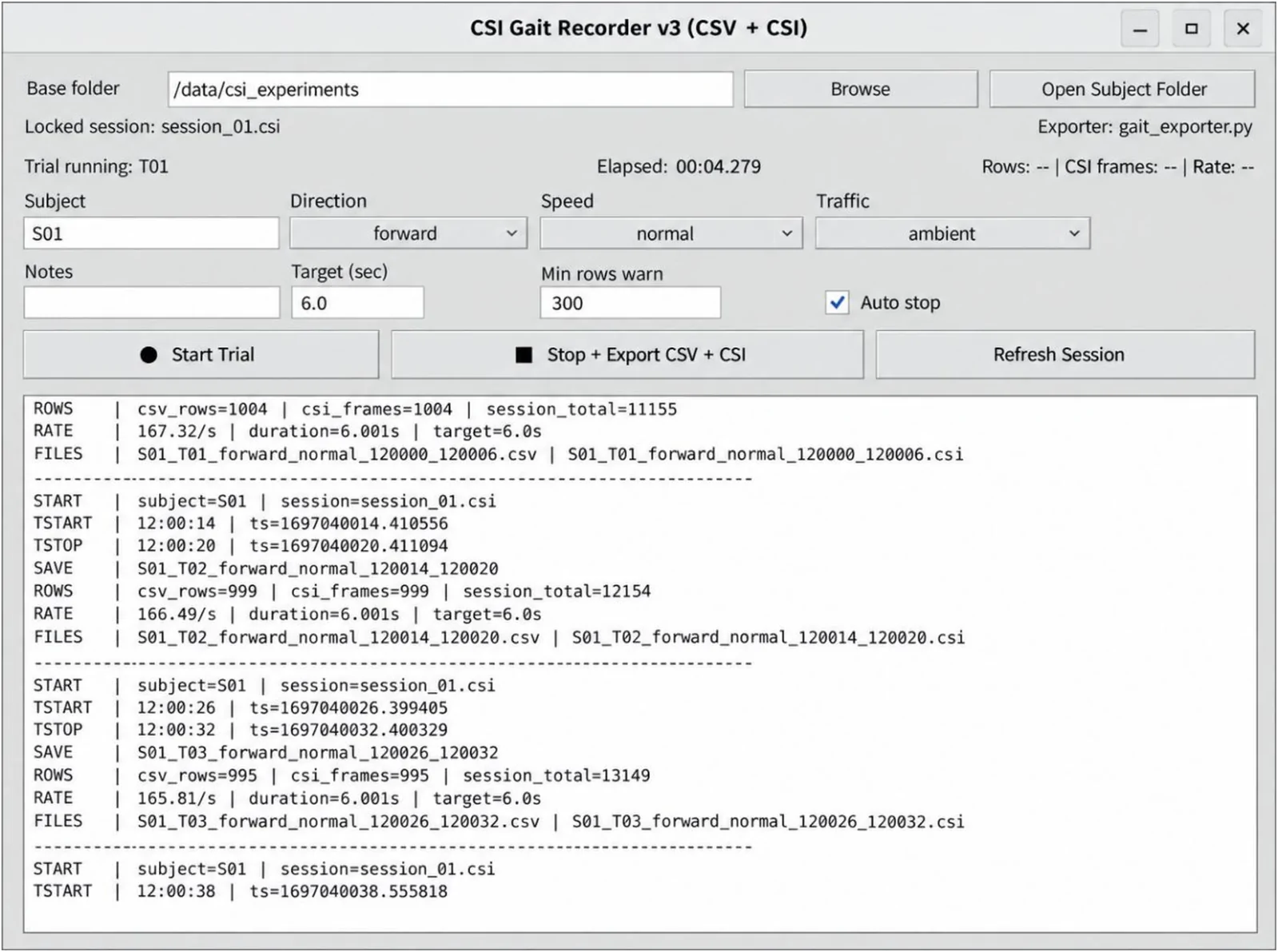
Custom graphical interface used for PicoScenes-based CSI trial control, labeling, session monitoring, and trial-level data export.

One raw CSV file was retained for every segmented trial. Each row represents a captured Wi-Fi packet and preserves the packet timestamp, RSSI, acquisition-configuration fields, and serialized interleaved in-phase and quadrature CSI payload described in the Data Description section. During acquisition, the trial-control interface generated session-specific filenames and operational labels. During preparation of the public release, these files were systematically mapped and renamed according to the standardized Sxx_Tyy_Direction_Condition.csv convention described in Table 2, without altering the underlying packet-level CSI measurements. This convention links each recording to its anonymous participant identifier, trial number, walking direction, and carrying condition. For example, S01_T01_L_R_normal.csv denotes the first normal left-to-right trial of participant S01. The raw CSV files constitute the immutable source layer of the release: packets rejected during later quality control were not repaired, interpolated in place, or written back to the original files. Packet filtering, CSI reconstruction, and temporal standardization were performed only when generating the processed representation and associated validation outputs.

### 4.4 Packet validation and CSI reconstruction

Each packet was checked against the retained acquisition configuration before CSI reconstruction. A packet was accepted when cbw = 80, numTones = 245, numRx = 2, numTx = 2, and data_len = 1960. Configuration-mismatched packets, non-finite timestamps, and payloads that could not be parsed into the expected structure were excluded from downstream processing while the source CSV remained unchanged. The expected payload length of 1,960 values corresponds to 245 tones × 2 receive channels × 2 transmit channels × 2 values for the in-phase and quadrature components. For every trial, the audit retained the raw packet count, accepted-packet count, rejected-packet count, and rejection percentage so that packet filtering remained traceable rather than implicit.

For each retained packet, adjacent serialized values were paired as in-phase and quadrature components. Complex CSI for stream *s*, subcarrier *k*, and packet time $t_{p}$ was reconstructed as:

(1) $$H_{s,k}\left(t_{p}\right)=I_{s,k}\left(t_{p}\right)+jQ_{s,k}\left(t_{p}\right),$$

where I and Q denote the paired in-phase and quadrature components and j is the imaginary unit. The 1,960 real-valued entries therefore yield 980 complex coefficients per valid packet. These coefficients were reshaped in their stored order into a 4 × 245 representation, corresponding to four generic CSI streams and 245 subcarriers per stream. The original flattening order was preserved throughout reconstruction; consequently, physical Tx-Rx antenna labels were not assigned where the mapping could not be independently verified.

### 4.5 Log-amplitude transformation and temporal standardization

Following packet validation and complex CSI reconstruction, the magnitude of each complex coefficient was computed and transformed to a logarithmic amplitude representation as:

(2) $$X_{s,k}\left(t_{p}\right)=\text{ln}\left(1+\left|H_{s,k}\left(t_{p}\right)\right|\right),$$

where $H_{s,k}\left(t_{p}\right)$ denotes the complex CSI coefficient for stream *s*, subcarrier *k*, and packet time $t_{p}$, while $X_{s,k}\left(t_{p}\right)$ denotes the corresponding log-amplitude value. The log1p transformation uses the natural logarithm to compress the dynamic range of the CSI magnitude while preserving the relative temporal variations of the signal. This representation was used as the basis of the released processed CSI rather than applying additional motion filtering or aggressive normalization.

Packet arrival times were not perfectly uniform and the number of valid packets varied slightly among trials. The retained packets were therefore ordered according to ts_sec, duplicate timestamps were removed, and the first valid timestamp was used as the temporal origin. For a trial extending from relative time 0to T, a uniformly spaced grid of 512 samples was defined as

(3) $$t_{n}=\frac{nT}{511},n=0,1,\ldots ,511.$$

The log-amplitude sequence of every stream and subcarrier was then linearly interpolated using the original packet timestamps onto this common temporal grid. Packet-level RSSI values were processed using the same temporal coordinates so that CSI and RSSI remained aligned after resampling. The resulting CSI representation therefore had a fixed dimension of 4 × 512 × 245 for every trial, while preserving the four-stream and full subcarrier structure of the validated signal.

This temporal standardization provides a consistent trial length for direct numerical analysis and model input without altering the original raw recordings. Importantly, temporal median filtering, Savitzky–Golay smoothing, static-component removal, or other motion-enhancement operations used in exploratory or model-specific analyses are not part of the canonical processed representation released with CSI-GaitCov. This separation keeps the released processed representation suitable for alternative filtering, normalization, feature-learning, and modeling strategies. A representative comparison of the raw and processed CSI representations is shown in Fig. 8.

**Fig. 8:**
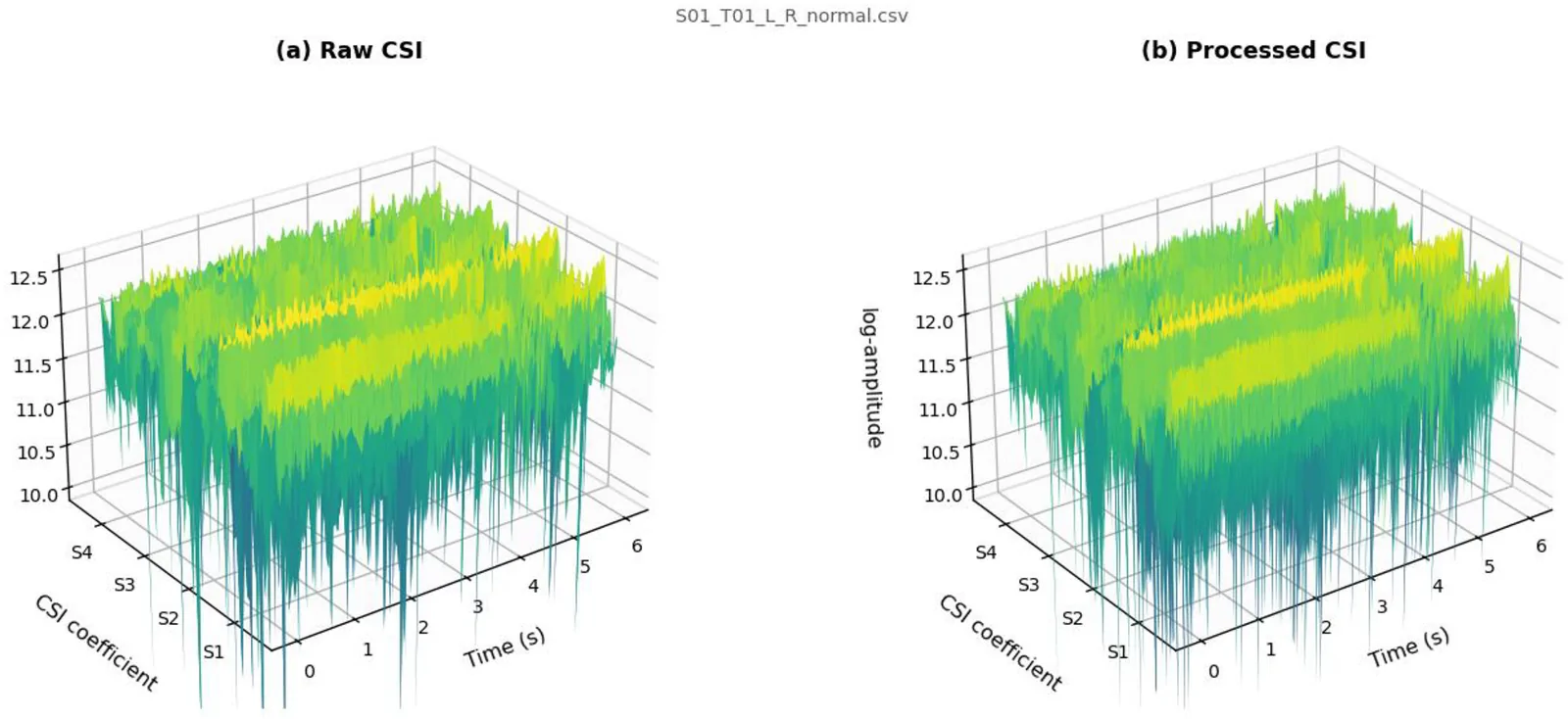
Representative raw and processed CSI log-amplitude representations from the same walking trial, showing the effect of temporal standardization.

### 4.6 Processed trial representation

The standardized output of each trial was stored as a compressed NumPy NPZ file using the same filename stem as the corresponding raw CSV recording. Each file contains three aligned arrays: x_logamp, t_sec, and rssi. The x_logamp array is stored as float32 with dimensions 4 × 512 × 245, corresponding to the four generic CSI streams, 512 standardized temporal samples, and 245 retained subcarriers. The t_sec array contains the associated 512-point relative-time grid, while the rssi array contains the RSSI sequence aligned to the same temporal coordinates. This fixed-size representation preserves the full four-stream and subcarrier structure of each validated trial while providing a common temporal dimension for direct computational reuse.

The processed NPZ files constitute the canonical processed layer of CSI-GaitCov and remain separate from the original packet-level CSV recordings. No model-specific normalization or dimensional reduction is embedded in these files; any additional transformations required for a particular benchmark or downstream analysis are applied independently after loading the released representation. This separation allows the same processed CSI data to support alternative feature extraction, filtering, normalization, and learning strategies without modifying the released trial-level arrays.

### 4.7 Four-stream feature extraction

In addition to the raw and processed CSI recordings, a compact trial-level feature representation was generated from the four standardized CSI streams and released as four_stream_features.csv. For each stream, 10 descriptive quantities were calculated: mean log-amplitude, standard deviation, median, interquartile range, dynamic root-mean-square (RMS), motion RMS, mean absolute temporal difference, temporal range, dominant frequency, and spectral entropy. Dynamic RMS was calculated after removal of the temporal median of each subcarrier, while dominant frequency and spectral entropy were obtained from the non-DC spectrum of the mean dynamic stream trace. The four streams therefore contributed 40 stream-wise descriptors.

To characterize relationships among the four CSI streams, the six unique pairwise Pearson correlation coefficients were also computed from their dynamic traces. Combining these with the 40 stream-wise descriptors produced a 46-dimensional feature vector for each trial. The feature-generation audit yielded one complete record for every processed trial, resulting in 2,500 rows in four_stream_features.csv. This derived table is distributed alongside the primary CSI arrays rather than as a replacement for them, and the corresponding stream-wise calculations are retained in the reproducible notebook so that the feature representation can be regenerated directly from the released NPZ files.

### 4.8 Technical validation and baseline benchmarking

The complete dataset was subjected to packet- and trial-level quality checks before benchmark evaluation. All 2,500 raw trial files were successfully read and all 2,500 corresponding processed NPZ files were generated, with no trial lost because of a processing failure. Across the complete collection, the mean number of captured packets was approximately 1,000.71 per trial, of which approximately 1,000.04 packets per trial satisfied the retained acquisition configuration. The mean trial duration was approximately 5.995 s, with a mean median packet rate of approximately 163.91 Hz, while the mean trial-level RSSI was approximately −55.3 dBm. Although 831 trials contained at least one packet that did not satisfy the retained configuration, the mean rejected-packet proportion across the dataset was only 0.0675%, and the maximum observed proportion was 3.4664%. No trial exceeded the 5% rejected-packet threshold. The principal full-dataset quality measures are summarized in Table 5.

**Table 5:** Technical validation summary of the complete CSI-GaitCov dataset.

Validation measure	Result
Raw trial files successfully read	2,500 / 2,500
Processed trial files generated	2,500 / 2,500
Processing failures	0
Mean raw packets per trial	≈ 1,000.71
Mean valid packets per trial	≈ 1,000.04
Mean trial duration	≈ 5.995 s
Mean median packet rate	≈ 163.91 Hz
Mean trial RSSI	≈ −55.3 dBm
Trials with ≥1 rejected packet	831
Mean rejected-packet percentage	0.0675%
Maximum rejected-packet percentage	3.4664%

Technical quality was additionally examined across the four experimental combinations of normal L→R, normal R→L, backpack L→R, and backpack R→L. As shown in Fig. 9, the distributions of valid packet count, trial duration, and mean RSSI were broadly comparable across these groups. This covariate-wise inspection complements the aggregate statistics in Table 5 and provides a direct check of acquisition consistency across the walking conditions represented in the dataset.

**Fig. 9:**
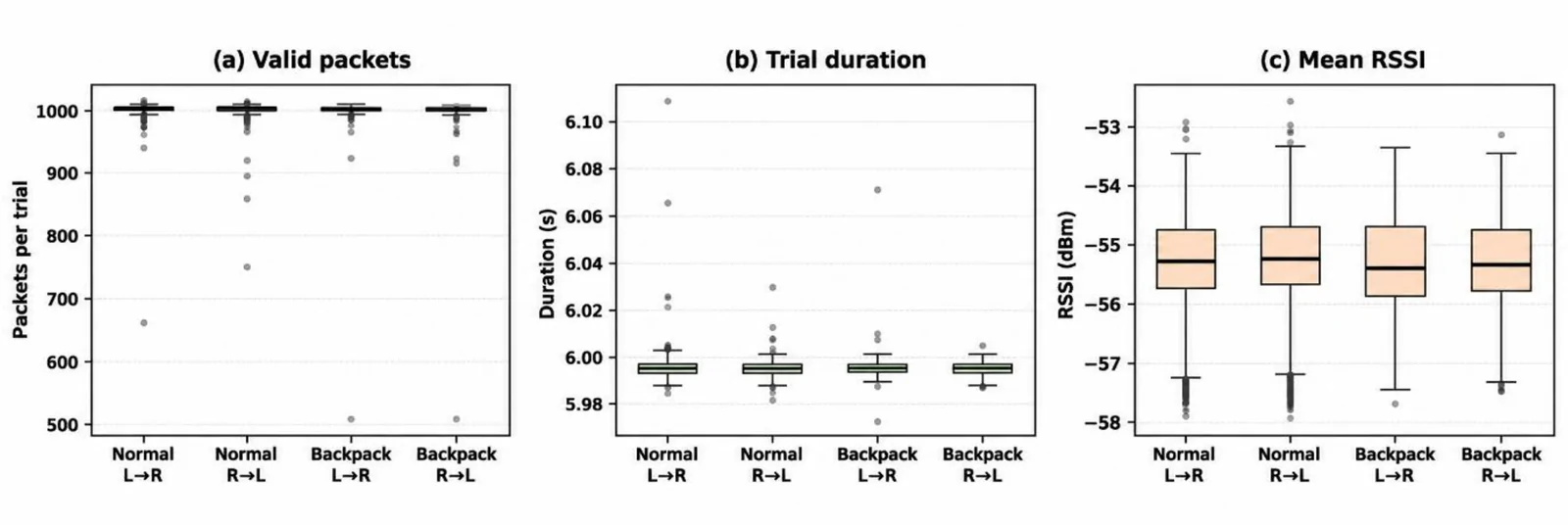
Technical quality across direction and carrying conditions.

To further verify the analytical usability of the released CSI data, three representative person-identification baselines were evaluated: an RBF-SVM, a 1D-CNN, and a 2D-CNN. The SVM used the 46-dimensional trial-level feature representation described above. The CNN baselines were constructed from the processed x_logamp arrays. Participant identifier was used as the classification target; walking direction and carrying-condition labels were not supplied to the models as predictive inputs.

The benchmark pipeline was implemented in Python, with the CNN experiments executed using PyTorch on a Windows workstation equipped with an AMD Ryzen 7 processor and an NVIDIA GeForce RTX 3050 GPU. CUDA-enabled GPU acceleration was used for CNN training and inference. The computational workflow, feature generation, predefined benchmark assignments, and evaluation procedures are retained in the released CSI_GaitCov_Pipeline.ipynb notebook to facilitate reproduction of the reported results.

For the standard closed-set benchmark, complete trials were assigned to 70% training, 15% validation, and 15% testing partitions, corresponding to 1,750 training, 375 validation, and 375 test trials. Both walking directions and both carrying conditions were represented within this mixed-condition evaluation. Trial assignment was performed before model fitting so that an individual walking trial could occur in only one partition.

Three stricter evaluations were subsequently performed to assess generalization under controlled covariate shifts. In the Normal train / Backpack test protocol, model training and selection used only normal-walking recordings, while all 500 backpack-carrying trials were reserved as the target test domain. For walking-direction transfer, one protocol used only left-to-right (L→R) trials for training and model selection and evaluated the resulting model on all 1,250 right-to-left (R→L) trials. The reverse protocol used R→L trials for training and model selection and all 1,250 L→R trials for testing. Consequently, the target carrying condition or target walking direction did not contribute to model development in the corresponding strict evaluation. The predefined assignments for these experiments are distributed in benchmark_splits.csv.

Additional precautions were applied to avoid direct train–test information leakage. For the SVM baseline, missing-value imputation, when required, and feature standardization were fitted using the training data only and subsequently applied to validation and test samples. For both CNN baselines, model selection was based on the validation partition and the held-out test trials were used only for final evaluation. The same 25 participant identities were intentionally represented across training and testing because the benchmark addresses closed-set person identification. However, the recordings themselves remained partitioned at the complete-trial level; the reported results should therefore be interpreted as identification of known participants from previously unseen walking trials rather than identification of previously unseen individuals.

Baseline performance is summarized in Table 6, where each cell reports Accuracy / Macro-F1. Accuracy and Macro-F1 were reported together so that overall classification success could be examined alongside performance averaged across the 25 participant classes. Under the standard mixed-condition test, the SVM achieved 93.33% / 93.28%, the 1D-CNN achieved 93.07% / 92.98%, and the 2D-CNN achieved 93.07% / 92.89%. Under the Normal train / Backpack test protocol, the corresponding results were 77.80% / 77.16%, 68.60% / 67.01%, and 70.60% / 67.68%. For L→R train / R→L test, the results were 87.84% / 87.73%, 84.16% / 83.77%, and 90.88% / 90.76%; for R→L train / L→R test, they were 85.68% / 85.47%, 84.16% / 84.21%, and 89.44% / 89.22%.

**Table 6:** Standard and strict baseline performance; cells report Accuracy / Macro-F1 (%).

Evaluation protocol	Test trials	SVM	1D-CNN	2D-CNN
Standard mixed	375	93.33 / 93.28	93.07 / 92.98	93.07 / 92.89
Normal train / Backpack test	500	77.80 / 77.16	68.60 / 67.01	70.60 / 67.68
L→R train / R→L test	1,250	87.84 / 87.73	84.16 / 83.77	90.88 / 90.76
R→L train / L→R test	1,250	85.68 / 85.47	84.16 / 84.21	89.44 / 89.22

The close agreement between Accuracy and Macro-F1 in the standard and direction-transfer evaluations indicates that the reported performance was not driven solely by a limited subset of participant classes. As an additional class-wise validation, normalized confusion matrices for the three models under the standard mixed-condition test are shown in Fig. 10. These matrices provide a direct view of identification performance across all 25 participant identities and complement the aggregate metrics reported in Table 6. The reproducible notebook also generates training-validation curves for the CNN baselines and 95% bootstrap confidence intervals for accuracy as supporting validation outputs, allowing the reported benchmark behavior to be examined beyond a single point estimate.

**Fig. 10:**
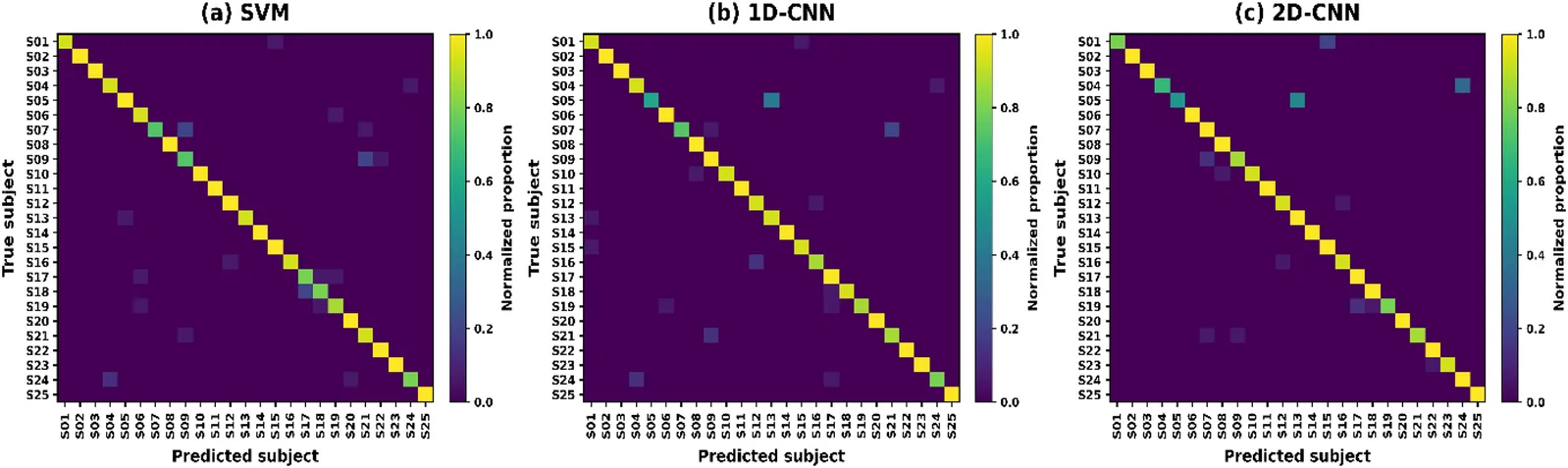
Row-normalized confusion matrices for (a) SVM, (b) 1D-CNN, and (c) 2D-CNN under the standard mixed-condition closed-set evaluation using the 70%/15%/15% trial-level train/validation/test split.

The baseline experiments are intended primarily to demonstrate the technical usability and reproducibility of CSI-GaitCov, rather than to establish state-of-the-art identification performance. The standard evaluation confirms that participant-discriminative information is retained in the released CSI representations, while the strict protocols provide reference results for assessing robustness to backpack carrying and changes in walking direction. The released repository also includes predefined benchmark splits, derived features, benchmark outputs, and the reproducible processing and validation notebook to support independent verification and future comparison.

## Limitations

CSI-GaitCov has several limitations that should be considered when reusing the dataset. The participant cohort consists of 25 male individuals aged 25–40 years, and therefore does not represent broader demographic variability such as female participants, younger or older age groups, or more diverse body characteristics. Data collection was performed in a single indoor laboratory using a fixed transmitter–receiver geometry and the same Wi-Fi hardware configuration throughout the acquisition campaign, so the dataset does not directly evaluate cross-room, cross-building, or cross-device variability. The walking protocol was intentionally controlled and includes only left-to-right and right-to-left walking under normal and backpack-carrying conditions; other potential gait variations, such as different walking speeds, heavier loads, turning trajectories, running, or simultaneous multi-person movement, were not included. In addition, the reported baseline experiments follow a closed-set person-identification setting, where the same 25 participant identities are represented across training and evaluation through different walking trials. Although the strict evaluation protocols quantify performance under backpack carrying and changes in walking direction, the reported results should not be interpreted as direct evidence of generalization to previously unseen individuals, substantially different environments, or different Wi-Fi sensing hardware. These limitations define the present scope of CSI-GaitCov and provide clear directions for future extensions of the dataset.

## Ethics Statement

The data collection involved human participants and was conducted in accordance with the ethical requirements of the host institution and the principles of the Declaration of Helsinki. Participation was voluntary, and all participants were informed about the purpose and procedures of the study before data acquisition. Written informed consent was obtained from each participant prior to recording. To protect participant privacy, personal identifiers were not included in the released dataset; anonymous participant codes (S01–S25) were used throughout the data files and associated metadata. Written consent for the use of participant photographs or visual representations in research dissemination and scholarly publication was obtained from the concerned participants. The study protocol was reviewed and approved by the Ethics Review Committee, Pabna University of Science and Technology, Pabna, Bangladesh, under Ref. No. PUST/ERC/115.

## CRediT authorship contribution statement

**Nakib Aman:** Conceptualization, Methodology, Supervision, Project administration, Formal analysis, Validation, Writing – review & editing. **Durjoy Kumar Dutta:** Methodology, Software, Investigation, Data curation, Formal analysis, Validation, Visualization, Writing – original draft, Writing – review & editing. **Md. Abdul Wadud:** Investigation, Data curation, Formal analysis, Validation. **Al Arian Ahmad:** Investigation, Data curation, Formal analysis, Validation.

## Data Availability

https://doi.org/10.5281/zenodo.22757555.
